# Suitable Promoter for DNA Vaccination Using a pDNA Ternary Complex

**DOI:** 10.3390/pharmaceutics16050679

**Published:** 2024-05-17

**Authors:** Tomoaki Kurosaki, Hiroki Nakamura, Hitoshi Sasaki, Yukinobu Kodama

**Affiliations:** 1 Graduate School of Biomedical Sciences, Nagasaki University, 1-7-1 Sakamoto, Nagasaki 852-8588, Japan; kurosaki@nagasaki-u.ac.jp (T.K.);; 2 Institute of Tropical Medicine, Nagasaki University, 1-12-4 Sakamoto, Nagasaki 852-8523, Japan; sasaki@nagasaki-u.ac.jp; 3 Department of Hospital Pharmacy, Nagasaki University Hospital, 1-7-1 Sakamoto, Nagasaki 852-8501, Japan

**Keywords:** promoters, γ-polyglutamic acid, gene delivery, polyethylenimine, pDNA

## Abstract

In this study, we evaluated the effect of several promoters on the transfection activity and immune-induction efficiency of a plasmid DNA (pDNA)/polyethylenimine/γ-polyglutamic acid complex (pDNA ternary complex). Model pDNAs encoding firefly luciferase (Luc) were constructed with several promoters, such as simian virus 40 (SV40), eukaryotic elongation factor 1 alpha (EF1), cytomegalovirus (CMV), and chicken beta actin hybrid (CBh) (pSV40-Luc, pEF1-Luc, pCMV-Luc, and pCBh-Luc, respectively). Four types of pDNA ternary complexes, each with approximately 145-nm particle size and −30-mV ζ-potential, were stably constructed. The pDNA ternary complex containing pSV40-Luc showed low gene expression, but the other complexes containing pEF1-Luc, pCMV-Luc, and pCBh-Luc showed high gene expression in DC2.4 cells and spleen after intravenous administration. After immunization using various pDNA encoding ovalbumin (OVA) such as pEF1-OVA, pCMV-OVA, and pCBh-OVA, only the pDNA ternary complex containing pCBh-OVA showed significant anti-OVA immunoglobulin G (IgG) induction. In conclusion, our results showed that the CBh promoter is potentially suitable for use in pDNA ternary complex-based DNA vaccination.

## 1. Introduction

The DNA vaccine concept is reportedly a promising therapeutic strategy for cancer and infection because it is safe, stable, and specific [1,2]. Melanoma is the most immunogenic of all solid cancers, and several melanoma-specific antigens have been reported [3]. However, a clinical trial has revealed that a DNA vaccine had little effect on melanoma in humans [4]. One promising approach to improving the effect of DNA vaccines is the development of DNA vaccine-delivery systems to antigen-presenting cells (APCs). In particular, dendritic cells (DCs) are good targets for DNA vaccines because they induce humoral and cell-mediated immunity [5,6]. Generally, DNA vaccines have been injected into humans by intradermal, intramuscular, or subcutaneous routes; however, it has been reported that intravenous administration of a DNA vaccine also showed a high immune-induction effect [7].

In a previous study, we developed a new DNA vaccine vector comprising a ternary complex of plasmid DNA (pDNA), polyethylenimine (PEI), and γ-polyglutamic acid (γ-PGA). The pDNA ternary complex selectively showed high gene expression in the marginal zone of the spleen without acute toxicity and liver toxicity [8]. The marginal zone is known to be rich in APCs such as DCs and the safe pDNA ternary complex is a suitable vector for intravenous administration of a DNA vaccine. Actually, intravenous administration of the pDNA ternary complex containing the melanoma DNA vaccine encoding ubiquitylated melanoma-related antigen induced a high immune response against melanoma cells; however, four injections were required to induce sufficient immunity.

To improve the immune-inducing effect of the pDNA ternary complex, high protein-expression efficiency in APCs is required. Promoters are essential factors for effective gene expression from pDNA and are strongly affected by the cell type [9,10,11]. Depending on the type of promoter, gene expression may reportedly be reduced by silencing [12,13]. Additionally, the promoter activity has also been reported to be affected by the type of gene-delivery vector [14]. In a previous study, we used the cytomegalovirus (CMV) promoter to evaluate the transfection activity of the pDNA ternary complex [8]; however, effective promoters for DCs using the pDNA ternary complex have not been identified.

In this study, we evaluated the efficiency of commonly used promoters such as the simian virus 40 (SV40), eukaryotic elongation factor 1 alpha (EF1), CMV, and chicken beta actin hybrid (CBh) promoters for gene expression in DCs. The SV40 promoter has been used for a long time and shows strong gene expression in baby hamster kidney fibroblasts, specifically BHK-21 cells [15]. The EF1 promoter stimulates high gene expression, particularly in fibroblasts and embryonic stem cells, and is tolerant to silencing caused by DNA methylation [16,17]. The CMV promoter is generally used, including by us, because it stimulates high gene expression in most cell lines [18]. The CBh promoter was recently developed to improve the gene expression of the chicken beta actin (CBA) promoter and shows strong and ubiquitous gene expression for central nervous system applications, equal to or better than those of the CMV or CBA promoters [19]. We constructed four pDNA ternary complexes each with a different pDNA and investigated suitable promoters for DNA vaccination.

## 2. Materials and Methods

### 2.1. Chemicals

Restriction enzymes (BglII, HindIII-HF, XbaI-HF, XhoI, SalI, and NcoI) were obtained from New England Biolabs Inc. (Ipswich, MA, USA). PicaGene control vector 2 encoding firefly luciferase (Luc) (pSV40-Luc) and PicaGene luminescence kit were purchased from Toyo B-net, Inc. (Tokyo, Japan). The pcDNA3.1 vector was obtained from Invitrogen Co. (Carlsbad, CA, USA), and pDRIVE5s-chEF1 was purchased from InvivoGen Co. (San Diego, CA, USA). The pCBh-vector and pcDNA3.1-ovalbumin (OVA) were synthesized from GenScript Co. (Piscataway, NJ, USA). Polyethylenimine (PEI; branched form, average molecular weight of 25,000) was purchased from Sigma-Aldrich Co. (Milwaukee, WI, USA). The γ-PGA was provided by Yakult Pharmaceutical Industry Co., Ltd. (Tokyo, Japan). All other chemicals were of the highest purity available.

### 2.2. Construction of pDNA

pSV40-Luc, pcDNA3.1-vector, and pCBh-vector were digested with BglII and HindIII-HF. The SV40 promoter was removed from pSV40-Luc, and a DNA ligation kit (Takara Bio Inc., Shiga, Japan) was used to subclone the CMV promoter and CBh promoter into the fragment to construct pCMV-Luc and pCBh-Luc. For the construction of pEF1-Luc, the SV40 promoter was removed from pSV40-Luc with XhoI and NcoI, and the EF1-promoter fragment using SalI and NcoI from pDRIVE5s-chEF1 was subcloned into the pSV40-Luc fragment. HindIII-HF and XbaI-HF were used to digest pEF1-Luc, pCMV-Luc, pCBh-Luc, and pcDNA3.1-OVA. Luciferase cDNA was removed and OVA cDNA was subcloned into each pDNA, followed by construction of pEF1-OVA, pCMV-OVA, and pCBh-OVA.

### 2.3. Preparation of the pDNA Ternary Complex

The pDNA ternary complex was prepared according to a previously reported method [8]. In brief, pDNA, PEI, and γ-PGA were mixed at a theoretical charge ratio of 1:8:6 for pDNA phosphate to PEI nitrogen to γ-PGA carboxylate in 5% glucose solution. We constructed pSV40-Luc/PEI/γ-PGA complex (pSV40-Luc complex), pEF1-Luc/PEI/γ-PGA complex (pEF1-Luc complex), pCMV-Luc/PEI/γ-PGA complex (pCMV-Luc complex), pCBh-Luc/PEI/γ-PGA complex (pCBh-Luc complex), pEF1-OVA/PEI/γ-PGA complex (pEF1-OVA complex), pCMV-OVA/PEI/γ-PGA complex (pCMV-OVA complex), and pCBh-OVA/PEI/γ-PGA complex (pCBh-OVA complex).

### 2.4. Physicochemical Properties of the Complex

A Zetasizer Nano ZS (Malvern Instruments Ltd., Malvern, UK) was used to measure the particle size and ζ-potential of each pDNA ternary complex. Particle sizes are shown as the ζ-average particle size.

### 2.5. Cell Culture

The mouse dendritic DC2.4 cell line was obtained from Sigma-Aldrich Co. The cells were incubated in Roswell Park Memorial Institute 1640 supplemented with 10% fetal bovine serum, 0.025 *w*/*v* % amphotericin B, 0.4 *w*/*v* % 2-mercaptoethanol, 1% Minimum Essential Medium Non-Essential Amino Acids Solution, 100 units/mL penicillin, and 0.01 *w*/*v* % streptomycin (culture medium) under a humidified atmosphere of 5% CO_2_ in air at 37 °C.

### 2.6. In Vitro Transfection Experiments

DC2.4 cells were placed into 24-well plates (Corning Inc., Corning, NY, USA) at a density of 1.0 × 10^4^ cells/well and cultivated in 500 µL culture medium. After 24 h of pre-incubation, the cells were treated with 0.25 μg/mL, 0.5 μg/mL, 1 μg/mL, and 2 μg/mL of each pDNA ternary complex for 2 h. After 22 h of incubation, the cells were washed with phosphate-buffered saline and then lysed in 100 µL lysis buffer (pH 7.8; 0.1 M Tris/HCl buffer containing 0.05% Triton X-100 and 2-mM ethylenediaminetetraacetic acid). A protein assay Coomassie brilliant blue solution (Nacalai tesque Inc., Kyoto, Japan) was used to determine the protein content of the lysate, with BSA as a standard, and a microplate reader (Infinite-200Pro M-Plex, Tecan Japan Co., Ltd., Kanagawa, Japan) at 595 nm was used to measure absorbance. A PicaGene luminescence kit and a luminometer (Lumat LB 9507; EG & G Berthold, Bad Wildbad, Germany) were used to determine luciferase activity. Luciferase activity is shown as relative light units (RLUs) per mg protein.

To assess the time-dependent transfection activity of the promoters, DC2.4 cells were treated with 2 μg/mL of each pDNA ternary complex for 2 h as described above. Luciferase activity was measured at 6, 12, 24, and 48 h after treatment.

### 2.7. Animals

All animal care and experimental procedures were performed according to the Guidelines for Animal Experimentation of Nagasaki University, with approval from the Institutional Animal Care and Use Committee. Male ddY mice and BALB/c mice (5 weeks old) were purchased from Japan SLC, Inc. (Shizuoka, Japan). After delivery, the mice were acclimatized to their new environment for ≥1 day before the experiments.

### 2.8. In Vivo Transfection Experiments

A 400 µL volume of pDNA ternary complex containing 10, 20, 40, and 80 mg of each pDNA was intravenously injected into each mouse through the tail vein to examine the transgene expression efficiency of the pDNA ternary complex. Six hours after injection with a pDNA ternary complex, the mice were sacrificed, and the lung, liver, and spleen were dissected. The tissues were washed twice with cold saline. The lysis buffer was added in a weight ratio of 3 mL/g for liver samples and 10 mL/g for other organ samples, and these samples were homogenized for 30 s by handy homogenizer (Omini International, Kennesaw, GA, USA). The resultant homogenates were centrifuged at 15,000 rpm (Kubota 3500; Kubota, Tokyo, Japan) for 5 min, and the associated supernatants were subjected to luciferase assays as described above. Luciferase activity is indicated as RLU per gram of tissue.

To assess the time-dependent transfection activity of the promoters, 400 µL of pDNA ternary complex containing 80 μg of each pDNA was intravenously injected. The mice were sacrificed, three tissues were dissected, and luciferase activity was measured at 6, 12, and 24 h after injection.

### 2.9. Vaccination

BALB/c mice were intravenously injected with the pEF1-OVA complex, pCMV-OVA complex, or pCBh-OVA complex twice at 2-week intervals through the tail vein. Two weeks after the last administration, the mice were sacrificed and serum was obtained. Anti-OVA IgG in the serum was determined by enzyme-linked immunosorbent assay (ELISA). The 100 µL of OVA solution (10 µg/mL, in 1 M sodium hydrogen carbonate) was added to each well of the ELISA plates (Thermo Fisher Scientific Inc., Waltham, MA, USA) and incubated overnight at 4 °C. The plates were washed with phosphate-buffered saline containing 0.05% Tween-20 (PBST) and incubated with 200 µL of blocking reagent N 102 (Nichiyu, Co., Ltd., Tokyo, Japan) for 6 h at 4 °C. The plates were washed and 100 µL aliquots of 1000-fold diluted serum samples were added to each well and incubated overnight at 4 °C. After washing with PBST, 100 µL of house radish peroxidase (HRP)-conjugated goat anti-mouse IgG (1:10,000) (Abcam, Cambridge, UK) was added to each well and incubated at room temperature for 1 h and then washed with PBST. TMB One solution (Promega, Madison, WI, USA) was used and prepared according to the manufacturer’s instructions. The reaction was then stopped at 15 min by addition of 1N hydrochloric acid. Absorbance was read at 450 nm using a microplate reader.

### 2.10. Statistical Analysis

Tukey’s test was used to perform multiple comparisons among the groups. *p* < 0.05 was accepted as indicating statistical significance.

## 3. Results

### 3.1. Physicochemical Properties of the pDNA Ternary Complex

We constructed four pDNAs encoding Luc with various promoters such as SV40, EF1, CMV, and CBh (pSV40-Luc, pEF1-Luc, pCMV-Luc, and pCBh-Luc, respectively). Various pDNA ternary complexes were constructed with these pDNAs, PEI, and γ-PGA. The measured particle size and ζ-potential of various pDNA ternary complexes are presented in Table 1 and were approximately 145 nm and −30 mV, respectively.

### 3.2. In Vitro Transfection Activity of the pDNA Ternary Complexes in DC2.4 Cells

**Figure 1:**
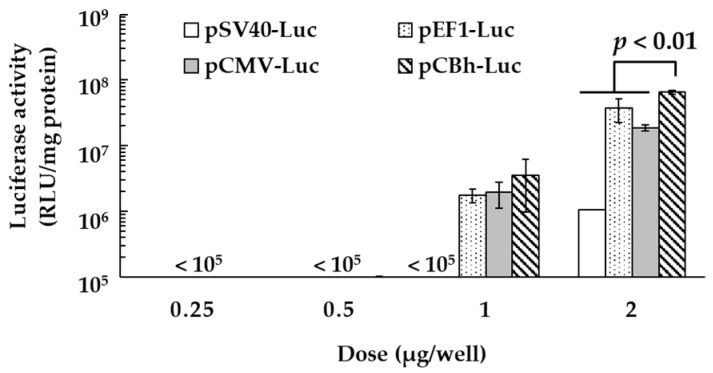
Transfection activity of the pDNA ternary complexes in DC2.4 cells. The transfection activity of the pDNA ternary complexes in DC2.4 cells was determined by measuring luciferase activity at 24 h post-treatment. Each bar represents the mean ± standard error (SE) (*n* = 3).

The transfection activity of each pDNA ternary complex was determined in DC2.4 cells at 24 h post-treatment. The transfection activity increased in a dose-dependent manner, and the luciferase activity was significantly higher for the pCBh-Luc complex than for the other pDNA ternary complexes at 2 μg/well (Figure 1, *p* < 0.01).

### 3.3. In Vitro Time-Dependent Transfection Activity of the pDNA Ternary Complexes in DC2.4 Cells

**Figure 2:**
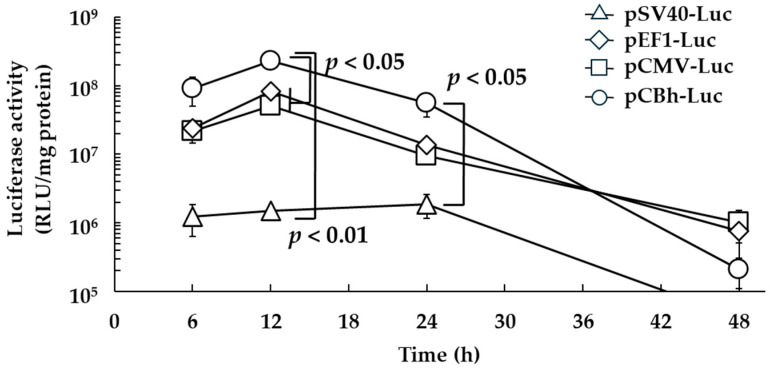
Time-dependent transfection activity of the pDNA ternary complex in DC2.4 cells. All pDNA ternary complexes containing 2 μg of pDNA were added to DC2.4 cells, and luciferase activity was measured at 6, 12, 24, and 48 h post-treatment. Each bar represents the mean ± SE (*n* = 3).

The luciferase activity, as an indicator of in vitro time-dependent transfection activity, was significantly higher for the pCBh-Luc complex pDNA ternary complex than for the other pDNA ternary complexes at 12 h post-treatment (Figure 2). In addition, the luciferase activity was significantly higher for the pCBh-Luc complex than for the pSV40-Luc complex at 24 h after administration. At 48 h post-treatment, the luciferase activity decreased to approximately 1% of the peak for all pDNA ternary complexes, and there was no remarkable difference in silencing.

### 3.4. In Vivo Transfection Activity of the pDNA Ternary Complexes

**Figure 3:**
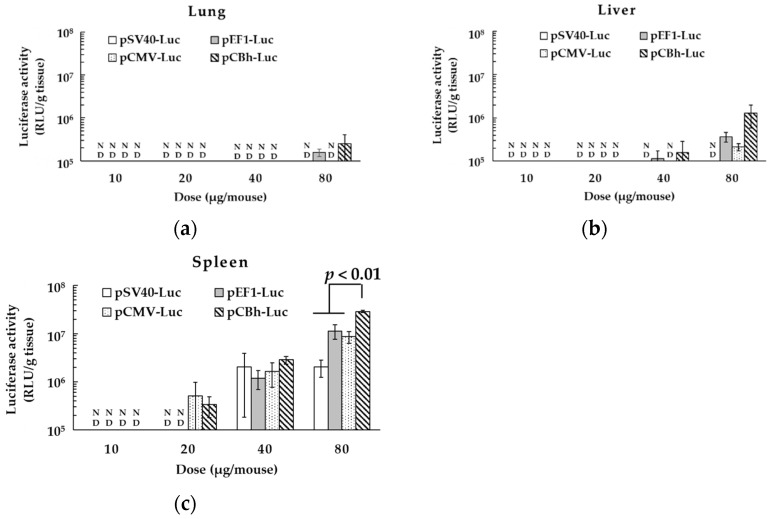
Transfection activity of the pDNA ternary complex in vivo. The in vivo transfection activity of the pDNA ternary complex was determined by measuring the luciferase activity of lung (**a**), liver (**b**), and spleen (**c**) homogenate at 6 h after intravenous administration. Each bar represents the mean ± SE (*n* = 3). “ND” means the sampled data is below measurable limit.

In a preliminary experiment, all pDNA ternary complexes containing 80 μg of pDNA showed luciferase activity below 1 × 10^5^ RLU/g tissue, which is a measurable limit in the kidney and heart after intravenous administration. Therefore, we determined the in vivo gene expression in the lung, liver, and spleen. In these organs, the transfection activity of each pDNA ternary complex increased dose dependently, and the spleen showed the highest gene expression among the tested tissues (Figure 3). In particular, the transfection activity in the spleen was significantly higher for the pCBh-Luc complex than for the other pDNA ternary complexes at a dose of 80 μg. However, in the liver and lung, there were no significant differences between these pDNA ternary complexes at all doses.

### 3.5. Time-Dependent Transfection Activity of the pDNA Ternary Complex In Vivo

**Figure 4:**
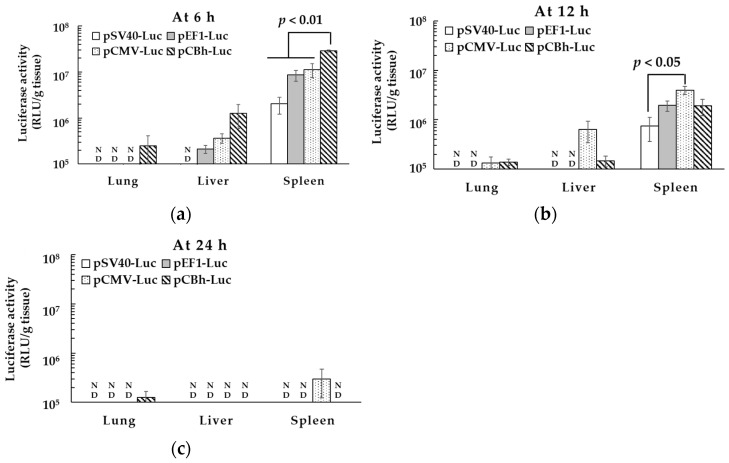
Time-dependent transfection activity of the pDNA ternary complex in vivo. All pDNA ternary complexes containing 80 μg of pDNA were administered, and the luciferase activity of each organ was measured at 6 (**a**), 12 (**b**), and 24 h (**c**) after administration. Each bar represents the mean ± SE (*n* = 3). “ND” means the sampled data is below measurable limit.

Time-dependent transfection activity of the pDNA ternary complex was determined in mice. Six hours after treatment, the pCBh-Luc complex showed significantly higher transfection activity in the spleen (Figure 4). After 12 h, the transfection activity was higher for the pCMV-Luc complex than for the pSV40-Luc in the spleen. In all pDNA ternary complexes, the luciferase activity was almost undetected at 24 h after administration. The SV40 promoter showed little gene expression in vivo; therefore, we chose the EF1, CMV, and CBh promoters for the next experiment.

### 3.6. Immune-Induction Effect of the pDNA Ternary Complex

**Figure 5:**
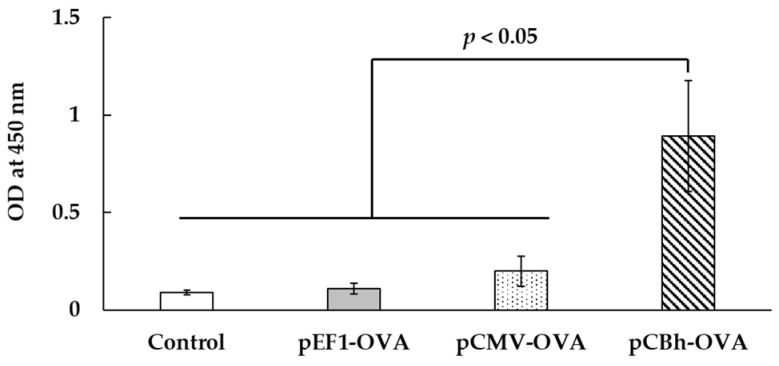
Anti-ovalbumin (OVA) immunoglobulin G (IgG) induction effect of the pDNA ternary complex. The pDNA ternary complexes with eukaryotic elongation factor 1 alpha (EF1), cytomegalovirus (CMV), or chicken beta actin hybrid (CBh) i.e., pEF1-OVA, pCMV-OVA, or pCBh-OVA, respectively, were intravenously administered to mice twice at 2-week intervals. Two weeks after the last administration, anti-OVA IgG in mouse serum was determined by enzyme-linked immunosorbent assay. Each bar represents the mean ± SE (*n* = 3).

The pDNA ternary complexes containing pEF1-OVA, pCMV-OVA, or pCBh-OVA were intravenously administered to mice twice, and anti-OVA immunoglobulin (Ig)G in the serum was measured (Figure 5). The pEF1-OVA and pCMV-OVA complexes showed little IgG induction; however, significantly higher anti-OVA IgG was observed in the pCBh-OVA complex than in the control and other complexes (*p* < 0.05).

## 4. Discussion

To improve the effect of DNA vaccines, effective DNA vaccine-delivery and high protein expression in APCs are required. In a previous study, we developed an efficient gene delivery vector, the pDNA ternary complex, which was constructed with pDNA, PEI, and γ-PGA. After intravenous administration, the pDNA ternary complex showed selective gene expression in the marginal zone of spleen, regardless of its anionic surface charge [8]. Kodama Y. et al. have reported that γ-PGA coated nanoparticles were taken by the cells via caveolae-mediated endocytosis [20]. Peng S.F. et al. have also reported that the ternary complex of chitosan, pDNA, and γ-PGA was recognized by γ-glutamyl transpeptidase in the cell membrane, resulting in a significant increase in its cellular uptake via caveolae-mediated endocytosis [21]. The marginal zone of spleen is known to be rich in APCs, such as DCs and macrophages [22]. Therefore, the γ-PGA coated complexes could be taken by the APCs in the spleen and show high gene expression in these mechanisms. Actually, pre-administration of clodronate liposomes decreased gene expression of γ-PGA coated complexes in the spleen. This result indicated that γ-PGA coated complexes were partly taken by the macrophages in the spleen [23]. Matsuo K. et al. have also reported that γ-PGA coated nanoparticles efficiently delivered entrapped antigenic proteins into DCs and presented antigens to DCs via major histocompatibility complex class I and II molecules [24]. Considering the information together, the pDNA ternary complex should be suitable for a DNA vaccine-delivery vector. In fact, the pDNA ternary complex containing a melanoma DNA vaccine induced an effective immune response against melanoma and inhibited melanoma lung metastasis [8]. Another study has shown that a pDNA ternary complex containing a malaria DNA vaccine stimulated high IgG induction against malaria, and 100% of the intravenously vaccinated mice survived after lethal challenge with *Plasmodium yoelii* [25]. However, three to four injections of pDNA ternary complex containing these DNA vaccines were needed to achieve sufficient immune induction.

To improve the effect of DNA vaccines, promoters in the pDNA should be optimized for the pDNA ternary complex. Promoters are sequences of DNA involved in transcriptional initiation that strongly affect the transcription efficiency of mRNA. Many promoters have been reported in the past [26]. For practical use of DNA vaccines, a promoter that shows high gene expression in vivo, especially in APCs, is required.

Therefore, we explored a suitable promoter for transfection using a pDNA ternary complex. We constructed four pDNAs that differed only in the promoter region and prepared four pDNA ternary complexes. There was no significant difference in particle size and ζ-potential between the four pDNA ternary complexes (Table 1).

In contrast, the in vitro transfection activity of the pDNA ternary complex was quite different for each promoter (Figure 1 and Figure 2). The pCBh-Luc complex had the highest transfection activity among the four pDNA ternary complexes at a dose of 2 μg/well at 6, 12, and 24 h after treatment. The luciferase activity was markedly decreased at 48 h after treatment in all promoters. Ochiai et al. have reported that exogenous DNA was inactivated by silencing and degradation in the cell after transfection [27]. The decrease of luciferase activity in this experiment could also be caused by silencing and degradation, and plasmid dilution in the cells might be partially involved.

There are few reports about promoter activity in DCs; however, the CMV promoter has reportedly shown high transfection activity in DCs [28,29]. The pCMV-Luc complex also showed high luciferase activity in the present study. The SV40 promoter has been used for a long time, but it has been reported to show lower transfection activity than that of the CMV promoter [30]. In fact, we observed little transfection activity of pSV40-Luc complex in DC2.4 cells. The EF1 promoter leads to transfection activity as high as that of the CMV promoter in mammalian cells. In particular, in fibroblasts and embryonic stem cells, the gene expression was higher with the EF1 promoter than with the CMV promoter [16]. In this experiment, the transfection activity of the EF1 promoter was as high as that of the CMV promoter, similar to the results in a previous report. In a previous study, the gene expressions were higher for the CMV immediate-early enhancer, chicken β-actin core promoter, and rabbit β-globin intron (CAG) promoter than for the CMV promoter in several cells [31,32]. The CBh promoter was constructed by improving the CAG promoter. In our study, we found that the transfection activity in DC2.4 was higher for the CBh promoter than for the CMV promoter. DC2.4 is a dendritic cell line which exhibits characteristic features of DCs; however, the cell line has been immortalized by transducing some oncogenes [33]. In future, transfection activities of these promoters should be determined in normal DCs.

Promoter activity sometimes differs between in vitro and in vivo conditions [34], and the pDNA ternary complex could be taken not only by DCs but also other types of cells in the spleen, lung, and liver after intravenous administration. Therefore, we administered pDNA ternary complexes intravenously and examined the transfection activity of each pDNA ternary complex in the lung, liver, and spleen. According to our previous report, the pDNA ternary complex led to high gene expression in the spleen [8]. In particular, the pCBh-Luc complex showed the highest transfection activity in the spleen 6 h after intravenous administration (Figure 3 and Figure 4). At 12 h post-transfection, luciferase activity was higher for the pCMV-Luc complex than for the pSV40-Luc complex. There was no significant difference in luciferase activity between the pCMV-Luc complex and pCBh-Luc or pEF1-Luc complex at 12 h. In all pDNA ternary complexes, the luciferase activity was almost undetected 24 h after administration.

We determined the immune-induction effect of the pDNA ternary complexes containing the EF1, CMV, and CBh promoters because the SV40 promoter showed little in vivo gene expression in this study. We used OVA as the model antigen and constructed pDNAs encoding OVA, such as pEF1-OVA, pCMV-OVA, and pCBh-OVA. The pDNA ternary complexes containing these pDNAs were administered to mice, and anti-OVA IgG in the serum was determined 2 weeks after the second administration. Then, significantly high anti-OVA IgG levels were only induced by pCbh-OVA, as shown in Figure 5. The high gene expression induced by pCBh-Luc in the early stage might have contributed to this high immune-induction effect.

In a previous report, the CAG promoter reportedly showed high gene expression in vivo [35]. It has been suggested that the CBh promoter should also show strong transfection activity in living organisms. Although the detailed mechanism of upregulated gene transcription by a hybrid promoter system, such as the CBh and CAG promoter, has not been fully elucidated [36], the CBh promoter had the best transfection activity and immune-induction efficiency among the four promoters for pDNA ternary complexes.

Unmethylated CPG-motifs in the pDNA are reported to be associated with an inflammatory reaction and a loss of gene expression [37,38]. Miura N. et al. have reported that removal of the CpG-motifs from the pDNA strongly boosted transfection efficacy of a lipid nanoparticle constructed by the authors [39]. In the future, the effect of CpG-motifs in the pDNA should be evaluated in the pDNA ternary complex. Additionally, it is worth exploring suitable introns, polyadenylation signals, enhancers, and untranslated regions, which are known to be important factors associated with improved transfection activity of pDNA [40,41,42].

In our study, we used four different promoters to compare the transfection and DNA vaccination efficiencies of pDNA ternary complexes. Among them, the CBh promoter was found to have good potential for use in a pDNA ternary complex.

## Figures and Tables

**Table 1:** Particle size and ζ-potential of pDNA ternary complexes.

Complexes	Size (nm)	ζ-Potential (mV)
pSV40-Luc	140.7 ± 3.2	−29.1 ± 0.7
pEF1-Luc	143.0 ± 2.6	−27.7 ± 0.7
pCMV-Luc	147.5 ± 3.6	−29.7 ± 0.4
pCBh-Luc	142.7 ± 1.2	−29.5 ± 1.5

Each datum represents the mean ± standard deviation.

## Data Availability

The data presented in this study are available on request from the corresponding author. The data are not publicly available due to legal restrictions.

## References

[1] Yang B., Jeang J., Yang A., Wu T.C., Hung C.F. (2014). DNA vaccine for cancer immunotherapy. Hum. Vaccines Immunother..

[2] Lopes A., Vandermeulen G., Preat V. (2019). Cancer DNA vaccines: Current preclinical and clinical developments and future perspectives. J. Exp. Clin. Cancer Res..

[3] Sang M., Wang L., Ding C., Zhou X., Wang B., Wang L., Lian Y., Shan B. (2011). Melanoma-associated antigen genes—An update. Cancer Lett..

[4] Rosenberg S.A., Yang J.C., Sherry R.M., Hwu P., Topalian S.L., Schwartzentruber D.J., Restifo N.P., Haworth L.R., Seipp C.A., Freezer L.J. (2003). Inability to immunize patients with metastatic melanoma using plasmid DNA encoding the gp100 melanoma-melanocyte antigen. Hum. Gene Ther..

[5] Zaneti A.B., Yamamoto M.M., Sulczewski F.B., Almeida B.D.S., Souza H.F.S., Ferreira N.S., Maeda D.L.N.F., Sales N.S., Rosa D.S., Ferreira L.C.S. (2019). Dendritic cell targeting using a DNA vaccine induces specific antibodies and CD4+ T cells to the dengue virus envelope protein domain III. Front. Immunol..

[6] Garu A., Moku G., Gulla S.K., Chaudhuri A. (2016). Genetic immunization with in vivo dendritic cell-targeting liposomal DNA vaccine carrier induces long-lasting antitumor immune response. Mol. Ther..

[7] McCluskie M.J., Brazolot Millan C.L., Gramzinski R.A., Robinson H.L., Santoro J.C., Fuller J.T., Widera G., Haynes J.R., Purcell R.H., Davis H.L. (1999). Route and method of delivery of DNA vaccine influence immune responses in mice and non-human primates. Mol. Med..

[8] Kurosaki T., Kodama Y., Muro T., Higuchi N., Nakamura T., Kitahara T., Miyakoda M., Yui K., Sasaki H. (2013). Secure splenic delivery of plasmid DNA and its application to DNA vaccine. Biol. Pharm. Bull..

[9] Qin J.Y., Zhang L., Clift K.L., Hulur I., Xiang A.P., Ren B.Z., Lahn B.T. (2010). Systematic comparison of constitutive promoters and the doxycycline-inducible promoter. PLoS ONE.

[10] Chung S., Andersson T., Sonntag K.C., Björklund L., Isacson O., Kim K.S. (2002). Analysis of different promoter systems for efficient transgene expression in mouse embryonic stem cell lines. Stem Cells.

[11] Fiszer-Kierzkowska A., Vydra N., Wysocka-Wycisk A., Kronekova Z., Jarzab M., Lisowska K.M., Krawczyk Z. (2011). Liposome-based DNA carriers may induce cellular stress response and change gene expression pattern in transfected cells. BMC Mol. Biol..

[12] Norrman K., Fischer Y., Bonnamy B., Wolfhagen Sand F., Ravassard P., Semb H. (2010). Quantitative Comparison of Constitutive Promoters in Human ES cells. PLoS ONE.

[13] Zuniga R.A., Gutierrez-Gonzalez M., Collazo N., Sotelo P.H., Ribeiro C.H., Altamirano C., Lorenzo C., Aguillon J.C., Molina M.C. (2019). Development of a new promoter to avoid the silencing of genes in the production of recombinant antibodies in Chinese hamster ovary cells. J. Biol. Eng..

[14] Martello E., Gillingham E.L., Phalkey R., Vardavas C., Nikitara K., Bakonyi T., Gossner C.M., Leonardi-Bee J. (2022). Systematic review on the non-vectorial transmission of Tick-borne encephalitis virus (TBEv). Ticks Tick-Borne Dis..

[15] Xiang Z.Q., Spitalnik S.L., Cheng J., Erikson J., Wojczyk B., Ertl H.C. (1995). Immune responses to nucleic acid vaccines to rabies virus. Virology.

[16] Mizushima S., Nagata S. (1990). pEF-BOS, a powerful mammalian expression vector. Nucleic Acids Res..

[17] Uchiyama K., Watanabe D., Hayasaka M., Hanaoka K. (2014). A novel imprinted transgene located near a repetitive element that exhibits allelic imbalance in DNA methylation during early development. Dev. Growth Differ..

[18] Boshart M., Weber F., Jahn G., Dorsch-Häsler K., Fleckenstein B., Schaffner W. (1985). A very strong Enhancer is located upstream of an immediate early gene of human Cytomegalovirus. Cell.

[19] Gray S.J., Foti S.B., Schwartz J.W., Bachaboina L., Taylor-Blake B., Coleman J., Ehlers M.D., Zylka M.J., McCown T.J., Samulski R.J. (2011). Optimizing promoters for recombinant adeno-associated virus-mediated gene expression in the peripheral and central nervous system using self-complementary vectors. Hum. Gene Ther..

[20] Kodama Y., Nakamura T., Kurosaki T., Egashira K., Mine T., Nakagawa H., Muro T., Kitahara T., Higuchi N., Sasaki H. (2014). Biodegradable nanoparticles composed of dendrigraft poly-L-lysine for gene delivery. Eur. Pharm. Biopharm..

[21] Peng S.F., Tseng M.T., Ho Y.C., Wei M.C., Liao Z.X., Sung H.W. (2011). Mechanisms of cellular uptake and intracellular trafficking with chitosan/DNA/poly(γ-glutamic acid) complexes as a gene delivery vector. Biomaterials.

[22] Kraal G. (1992). Cells in the marginal zone of the spleen. Int. Rev. Cytol..

[23] Kodama Y., Tokunaga A., Hashizume J., Nakagawa H., Harasawa H., Kurosaki T., Nakamura T., Nishida K., Nakashima M., Hashida M. (2021). Evaluation of transgene expression characteristics and DNA vaccination against melanoma metastasis of an intravenously injected ternary complex with biodegradable dendrigraft poly-L-lysine in mice. Drug Deliv..

[24] Matsuo K., Ishii Y., Matsuo K., Yoshinaga T., Akashi M., Mukai Y., Yoshioka Y., Okada N., Nakagawa S. (2010). The utility of poly(γ-glutamic acid) nanoparticles as antigen delivery carriers in dendritic cell-based cancer immunotherapy. Biol. Pharm. Bull..

[25] Cherif M.S., Shuaibu M.N., Kurosaki T., Helegbe G.K., Kikuchi M., Yanagi T., Tsuboi T., Sasaki H., Hirayama K. (2011). Immunogenicity of novel nanoparticle-coated MSP-1 C-terminus malaria DNA vaccine using different routes of administration. Vaccine.

[26] Haruyama N., Cho A., Kulkarni A.B. (2009). Overview: Engineering transgenic constructs and mice. Curr. Protoc. Cell Biol..

[27] Ochiai H., Harashima H., Kamiya H. (2006). Silencing of exogenous DNA in cultured cells. Biol. Pharm. Bull..

[28] Papagatsias T., Rozis G., Athanasopoulos T., Gotch F., Dickson G., Patterson S. (2008). Activity of different vaccine-associated promoter elements in human dendritic cells. Immunol. Lett..

[29] Moulin V., Morgan M.E., Eleveld-Trancikova D.E., Haanen J.B.A.G., Wielders E., Looman M.W.G., Janssen R.A.J., Figdor C.G., Jansen B.J.H., Adema G.J. (2012). Targeting dendritic cells with antigen via dendritic cell-associated promoters. Cancer Gene Ther..

[30] Zarrin A.A., Malkin L., Fong I., Luk K.D., Ghose A., Berinstein N.L. (1999). Comparison of CMV, RSV, SV40 viral and Vlambda1 cellular promoters in B and T lymphoid and non-lymphoid cell lines. Biochim. Biophys. Acta.

[31] Dou Y., Lin Y., Wang T.Y., Wang X.Y., Jia Y.L., Zhao C.P. (2021). The CAG promoter maintains high-level transgene expression in HEK293 cells. FEBS Open Bio.

[32] Seita Y., Tsukiyama T., Azami T., Kobayashi K., Iwatani C., Tsuchiya H., Nakaya M., Tanabe H., Hitoshi S., Miyoshi H. (2019). Comprehensive evaluation of ubiquitous promoters suitable for the generation of transgenic cynomolgus monkeys. Biol. Reprod..

[33] Shen Z., Reznikoff G., Dranoff G., Rock K.L. (1997). Cloned dendritic cells can present exogenous antigens on both MHC class I and class II molecules. J. Immunol..

[34] Matouk C.C., Marsden P.A. (2008). Epigenetic regulation of vascular endothelial gene expression. Circ. Res..

[35] Kevin H., Elien De S., Nick G., Paul D. (2018). Prolonged in vivo expression and anti-tumor response of DNAbased anti-HER2 antibodies. Oncotarget.

[36] Watanabe M., Sakaguchi M., Kinoshita R., Kaku H., Ariyoshi Y., Ueki H., Tanimoto R., Ebara S., Ochiai K., Futami J. (2014). A novel gene expression system strongly enhances the anticancer effects of a REIC/Dkk-3-encoding adenoviral vector. Oncol. Rep..

[37] Hodges B.L., Taylor K.M., Joseph M.F., Bourgeois S.A., Scheule R.K. (2004). Long-term transgene expression from plasmid DNA gene therapy vectors is negatively affected by CpG dinucleotides. Mol. Ther..

[38] Hyde S.C., Pringle I.A., Abdullah S., Lawton A.E., Davies L.A., Varathalingam A., Nunez-Alonso G., Green A.M., Bazzani R.P., Sumner-Jones S.G. (2008). CpG-free plasmids confer reduced inflammation and sustained pulmonary gene expression. Nat. Biotechnol..

[39] Miura N., Shaheen S.M., Akita H., Nakamura T., Harashima H. (2015). A KALA-modified lipid nanoparticle containing CpG-free plasmid DNA as a potential DNA vaccine carrier for antigen presentation and as an immune-stimulative adjuvant. Nucleic Acids Res..

[40] Choi T., Huang M., Gorman C., Jaenisch R. (1991). A generic intron increases gene expression in transgenic mice. Mol. Cell Biol..

[41] Huynh C.Q., Zieler H. (1999). Construction of modular and versatile plasmid vectors for the high-level expression of single or multiple genes in insects and insect cell lines. J. Mol. Biol..

[42] Lee M., Choi D., Choi M.J., Jeong J.H., Kim W.J., Oh S., Kim Y.H., Bull D.A., Kim S.W. (2006). Hypoxia-inducible gene expression system using the erythropoietin enhancer and 3′-untranslated region for the VEGF gene therapy. J. Control Release.

